# Tooth-Supporting Hard Tissue Regeneration Using Biopolymeric Material Fabrication Strategies

**DOI:** 10.3390/molecules25204802

**Published:** 2020-10-19

**Authors:** Min Guk Kim, Chan Ho Park

**Affiliations:** 1 Department of Dental Science, Graduate School, Kyungpook National University, Daegu 41940, Korea; minguk.kim@knu.ac.kr; 2 Department of Dental Biomaterials, School of Dentistry, Kyungpook National University, Daegu 41940, Korea; 3 Institute for Biomaterials Research and Development, Kyungpook National University, Daegu 41940, Korea

**Keywords:** biopolymers, alveolar bone, cementum, tissue engineering, regenerative medicine, fabrication, periodontal tissues

## Abstract

The mineralized tissues (alveolar bone and cementum) are the major components of periodontal tissues and play a critical role to anchor periodontal ligament (PDL) to tooth-root surfaces. The integrated multiple tissues could generate biological or physiological responses to transmitted biomechanical forces by mastication or occlusion. However, due to periodontitis or traumatic injuries, affect destruction or progressive damage of periodontal hard tissues including PDL could be affected and consequently lead to tooth loss. Conventional tissue engineering approaches have been developed to regenerate or repair periodontium but, engineered periodontal tissue formation is still challenging because there are still limitations to control spatial compartmentalization for individual tissues and provide optimal 3D constructs for tooth-supporting tissue regeneration and maturation. Here, we present the recently developed strategies to induce osteogenesis and cementogenesis by the fabrication of 3D architectures or the chemical modifications of biopolymeric materials. These techniques in tooth-supporting hard tissue engineering are highly promising to promote the periodontal regeneration and advance the interfacial tissue formation for tissue integrations of PDL fibrous connective tissue bundles (alveolar bone-to-PDL or PDL-to-cementum) for functioning restorations of the periodontal complex.

## 1. Introduction

### 1.1. Tooth-Supportive Structures; Periodontal Tissues

**Figure 1:**
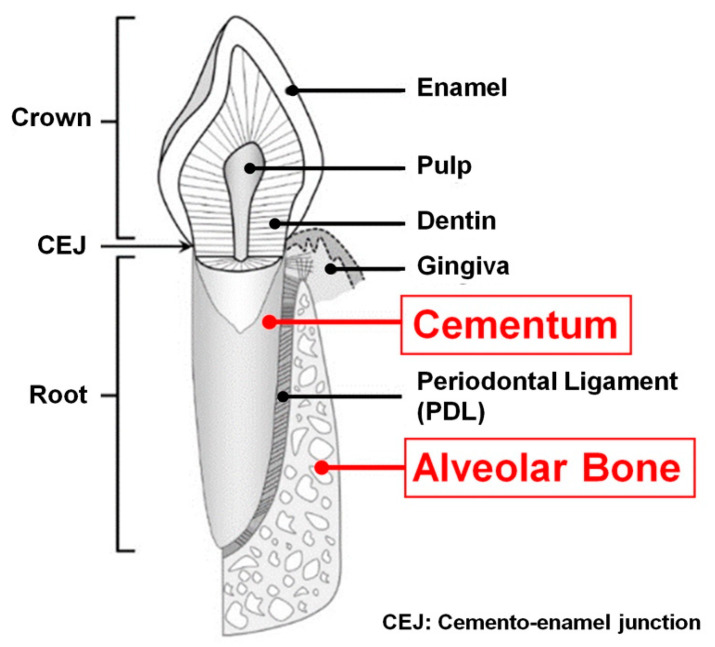
Schematic anatomy of tooth and periodontal tissues (tooth-supporting complex). Alveolar bone and cementum (red-colored terminologies) are typical mineralized tissues of the periodontal complex. Adapted with permission from the reference [5].

The fundamental function of periodontal tissues is to support the teeth during external stimulation, such as mastication and occlusion, as well as position the teeth in the alveolar bone sockets [1,2]. The periodontal complexes are composed of the alveolar bone: mineralized tissue to support the tooth and tooth-supporting tissue; the periodontal ligament (PDL): the fibrous connective soft tissues with the specific angulations to transmit mechanical stimulation; the cementum: the mineralized layer on the tooth-root surface that anchors the PDL fibrous bundles to the tooth-dentin surface; and the gingiva: the soft tissue on the alveolar bone crests that prevents microbiome invasion into the periodontal complexes [1,3,4,5]. Recently, many different studies have concentrated on engineered PDL formations with perpendicular or oblique orientations to the tooth-root surface using biopolymer-based scaffolds [6,7] or PDL cell sheet technologies [8,9,10]. PDL tissues within 200–300 µm interfaces are situated between two different mineralized tissues, such as the alveolar bone and cementum, for mastication-mediated adaptations or biomechanical transmission [1,5,11]. PDL Sharpey’s fibers, which are the end terminals of collagenous PDL bundles and facilitate the insertion of PDL fibers into mineralized tissues (bone and cementum), can generate biochemical responses [12,13,14]. Therefore, the mineralized tissues that anchor Sharpey’s fiber bundles are important to structuralize the periodontal complex (alveolar bone–PDL–cementum) as tooth-supporting architectures (Figure 1) [5,13].

The mandible has two different bone tissues: basal bone and alveolar bone, and they can be physiologically recognized based on mineral densities and biochemical evaluations [15,16]. The basal bone is formed in the fetus prior to tooth development, as part of the skeleton, and can be existed for the oral maxillofacial structure retention even though alveolar bone is completely resorbed after tooth loss [17]. The main roles of the basal bone in the cranial–oral–maxillofacial complex are having various facial muscle attachments for movement and containing mandibular canals for the alveolar nerve and vein-artery systems, rather than supporting the teeth [17]. The alveolar bone in the periodontal complex is the part of the maxilla or mandible for the teeth and is a dynamic tissue for adapting architectures and generating responses against biomechanical stimulation from tooth locomotion, mastication, and occlusion, which can be transmitted from the teeth and PDLs [16]. Therefore, tooth movements are significantly associated with the rapid remodeling (formation–resorption) of alveolar bone tissues and are continuously performed to generate the homeostatic balance between resorption by osteoclastic cells and apposition by osteoblastic cells [18,19]. As a result of bone remodeling processes, the mineral density of the alveolar bone surrounding the teeth can have a lower mechanical stiffness than basal bone structures [18,19]. Moreover, interfacial structures, which are the mineralized tissues on the socket surfaces between the alveolar bone and the PDL regions, can be used as anchorage of Sharpey’s collagenous fiber bundles of the PDLs to form hierarchical architectures for systematic tooth-supporting functions [1,3,20].

Cementum is the mineralized tissue layer on the tooth-root surface and plays the critical role of PDL functionalization by anchoring collagenous Sharpey’s fiber bundles to tooth-root dentin surfaces [14,21,22,23,24]. There are two major classifications of cementum: the acellular extrinsic fiber (acellular) cementum and the cellular intrinsic fiber (cellular) cementum [25,26,27]. Acellular cementum is found on the cervical and mid-portion of tooth roots with thin layers (50–200 µm thickness) and plays a pivotal role in attaching principal fibers (Sharpey’s fibers) onto tooth-root dentin surfaces [14,28]. It has limited cementogenic potential and receives biological supplements through the PDL interfaces due to the absence of vasculatures in the cementum [14,28,29,30]. Hence, acellular cementum–PDL integration can systematically transmit mechanical forces by mastication or occlusion [3]. Cellular cementum is made up of cementocyte-embedded mineralized layers on the apical portion of the tooth-root surfaces with various thicknesses in different teeth, such as incisors (400–600 µm), canines (approximately 500 µm), premolars (300–1000 µm), and molars (700–1500 µm) [25,31,32]. Because of cementogenesis, cementoblasts are considered to be a unique phenotype, whereas positional osteoblasts are not [33].

### 1.2. Periodontal Destruction by Periodontitis and Therapeutic Strategies

Periodontitis (or periodontal disease) is one of the most common inflammatory infectious diseases affecting humans [34,35] and severe forms lead to tooth loss [13,36,37]. Fundamentally, periodontal disease is initially triggered by bacteria or bacterial metabolic products; however, the results are mainly inflammatory responses and the subsequent destruction of the systematically interconnected tooth-supporting structure, i.e., alveolar bone–PDL–cementum [36,38,39,40]. In addition to progressive loss of periodontal complexes, periodontal disease is suspected as a co-factor in systemic diseases, such as diabetes mellitus, cardiovascular, liver, or lung disease [40,41].

Conventional therapeutic approaches have focused on eliminating inflammatory sources, preventing disease progression, and regenerating periodontia using osteoconductive or osteoinductive biopolymeric materials, which commonly contain bioactive signaling molecules [42,43,44,45,46]. However, periodontal tissue formations are extremely limited and unpredictable in terms of the regeneration process due to structural complications, hierarchical constructs with micron-scaled dimensions, and elaborate tissue integrations for a functioning restoration [3,47]. To overcome these limitations, various biologically achievable strategies in the regenerative medicine of periodontal tissues have recently been proposed for the restoration of lost tooth-supporting structures and multiple tissue integrations [21,48,49,50,51,52]. Moreover, computer-aided-design (CAD) technology has been utilized to design and manufacture biopolymeric scaffolding systems to attempt the spatiotemporal regeneration of periodontal tissues with structural integrations for tooth-supporting function restorations in preclinical situations [53,54]. Although the study outcomes of periodontal tissue engineering treatments have been tremendously promising for novel therapeutic developments, such as mineralized tissue formations with dimensional controls for spatial compartmentalization, and complete integrations of hard and soft tissues like alveolar bone–PDL or PDL–cementum for functioning restorations in periodontal complexes, many challenges remain. In this review, we outline the recently proposed biomaterial fabrication techniques for tooth-supporting hard tissue regeneration, such as the alveolar bone and the cementum surrounding the teeth.

## 2. Natural Biopolymers for Periodontal Hard Tissue Regeneration

### 2.1. Collagen and Denatured-Collagen (Gelatin) Matrices

Collagen, as a natural polymer, is the most abundant extracellular matrix (ECM) protein; it is present within mineralized tissues, connective fibrous tissues, and various organs [55,56,57] and provides essential structures to support cell–tissue morphogenesis [58,59,60]. The interaction between collagen matrices and cells–tissues can produce various signaling molecules to regulate cell adhesion, proliferation, differentiation, and migration [61] and physiologically promote tissue development [58,62]. The collagen has typical triple-helical structures of three polypeptide chains, and their supramolecular organization can be characterized by its extreme complexity and the various functions with tissue-adaptable forms [63,64]. Of the many different types of collagen, type-I collagen is the most abundant and forms the main structures of mineralized tissues as well as soft tissues such as ligaments, including PDLs or gingiva fibrous tissues in dental and craniofacial tissue complexes [2,38,65]. In particular, the formation of calcium-phosphate crystals within the collagen fibrils, as the mineralized tissue develops, endows them with mechanical properties such as load bearing ability, tensile strength, and torsional stiffness [66,67,68].

Regarding their biological aspects, low antigenicity and high biocompatibility are extremely important for implantable biomaterials [69,70,71], and the biodegradability of type-I collagen matrices (or fibril structures) facilitates the bone remodeling process by osteoblastic and osteoclastic cells [72,73]. Moreover, collagen matrices can incorporate various growth factors or biologics [74,75] and so can be fabricated to deliver bioactive molecules for mineralized tissue regeneration in the form of gels, nanofibrous membranes, and scaffolds [58,76,77,78]. The denatured collagen matrix gelatin has been widely utilized as a drug delivery system [79,80] and in craniofacial bone tissue engineering in preclinical or clinical scenarios [81,82]. Compared with collagen, the biodegradability and mechanical properties of gelatin materials can easily be controlled using various cross-linking methods [83,84,85], and for this reason, different types of gelatin matrices have been developed as carriers for biomolecule-release systems [86,87] and in bone tissue regeneration scaffolds [88,89].

### 2.2. Fibrin Matrices

The fibrin matrix (or fibrin) is a typical natural biopolymer, which can be formed by fibrinogen polymerization with thrombin for blood coagulation [90,91]. Briefly, fibrin can be created by the polymerization of soluble fibrinogen monomers, which are formed after the specific cleavage of fibrinogen by the serine protease thrombin [92]. Interestingly, the concentration of thrombin is the key parameter to provide various physical properties, such as stiffness or plasticity with structural specifications, in the fibrin matrix with an insoluble 3D network [91]. The concentration of thrombin and fibrinogen can act to model fibrin filament networks and fiber densities to modify matrix elasticity and pore geometries [90,91,93,94].

Moreover, fibrin matrices can easily be fabricated by controlling the concentration of thrombin molecules, which can regulate interactions of the cell/tissue–fibrin matrix for enzymatic biodegradability or tissue regeneration as well as the main functions of fibrin matrices, such as physiological hemostasis and initial tissue wound sealing [90,91]. Fibrin degradation (fibrinolysis) is mainly regulated by the cell surface-associated plasmin, which is a complex of soluble plasminogen and plasminogen activators [90,95]. On the basis of the degradation mechanism, fibrinolysis may be the key player in the temporal and spatial arrangement to promote bone tissue regeneration and tissue infiltration into defects [96,97,98]. Therefore, controllable biodegradability and biological compatibility have practical potential for both 3D scaffolds and the characterization biopolymers for drug delivery systems with additional biologics, such as cells, enzymes, and growth factors, in order to promote hard tissue regeneration [99,100,101]. As a result of their relatively poor mechanical properties, and despite their excellent biological potential, composite biomaterials like fibrin-synthetic polymer materials or fibrin-inorganic materials have been recently developed for use in 3D architectures and have been investigated to improve their biomechanical strength for musculoskeletal and dental tissue engineering [102,103]. In addition to scaffolding systems, fibrin can be manipulated in the form of fibrin microbeads to encapsulate stem cells [104,105] and be used in fibrin-coating applications on more mechanical-stable materials [106,107] and injectable fibrin hydrogels [92,103,107,108,109].

## 3. Synthetic Biopolymers for Periodontal Hard Tissue Regeneration

### 3.1. Poly Lactic-co-glycolic Acid (PLGA)

Regarding the synthetic biopolymers, poly lactic-co-glycolic acid (PLGA) has been approved by the US Food and Drug Administration (US-FDA) and is widely utilized in various clinical applications in tissue engineering and regenerative medicine due to its biocompatibility, nontoxicity, mechanical properties, and fabricable biodegradability [110,111,112]. On the basis of its properties, many applications have been investigated, such as resorbable sutures, wound healing materials, 3D scaffolds for tissue engineering, and drug delivery systems with controlled release mechanisms [69,113]. PLGA is an aliphatic degradable biopolymer and random copolymer of poly lactic acid (PLA) and poly glycolic acid (PGA) with lactic acid/glycolic acid (LA/GA) ratios [111,114]. Theoretically, PGA has more crystalline structures with a higher hydrophilicity than PLA, which has the methyl group on a backbone chain. Therefore, the molecular ratio of monomers (LA/GA) is one of the major parameters to critically determine the biodegradation rates and characterize the mechanical properties of copolymerized PLGAs [115]. For example, the PLGA 75:25 (75% LA and 25% GA) demonstrated more amorphous structures, a lower hydrophilicity, and slower degradation than PLGA 50:50 [116,117]. As a result of the higher hydrophilicity of the amorphous PLGA copolymer with a higher ratio of PGA (e.g., PLGA 50:50), more water absorption is possible which results in faster hydrolytic degradation rates [116,118]. Therefore, the hydrolysis rates may be dependent on different ratios of LA/GA during fabrication, and the copolymerization process could be used to manufacture optimal drug delivery systems with degradation kinetics [119,120]. In addition, highly crystalline PGA has greater mechanical properties than PLA, including strength, toughness, and elasticity, so copolymer PLGA could also be modified to obtain the required mechanical or physical properties [119,121].

Rather than 3D scaffolding architectures for bone regeneration, the PLGA biopolymer has been mainly considered and utilized to create nano-/micro-particles to deliver biologics such as growth factors, proteins, drugs, or cells to target tissues [78,111,122,123,124,125,126]. Moreover, PLGA-based, resorbable barrier membranes have been manufactured for guided bone regeneration (GBR) techniques, which prevent soft tissue infiltration and induce bone formation in defect sites in periodontal tissue engineering [118,127,128,129,130].

### 3.2. Poly-ε-caprolactone (PCL)

Poly-ε-caprolactone (PCL) has been extensively investigated for medical applications in mineralized tissue and soft tissue engineering, because it is biocompatible and biodegradable (or hydrolytic-degradable) [131,132,133]. Since its approval by the US-FDA, PCL has been utilized for urgent specific applications in clinical situations, for example, as an additive manufacturing material for periodontal scaffolds or tracheal splints [134,135,136]. PCL is a semi-crystalline biopolymer and has a lower melting temperature (T_m_ = ~60 °C) than PLA (T_m_ = 130~180 °C), PGA (T_m_ = ~230 °C), or PLGA (depending on copolymerization with specific ratio of LA/GA) and a higher solubility to various organic solvents [131,133,137]. On the basis of its molecular characteristics, PCL is easily processible to achieve the required properties for various pharmaceutical or medical applications, such as great elasticity, viscoelasticity, ductility, and elongation at break, even though it has relatively poor mechanical stiffness [138,139,140]. In particular, as a result of the low glass transition temperature (T_g_ = −60 °C), the ductility of PCL (in contrast to the brittleness of PLA, PGA, and PLGA) can be complemented with a similar viscoelasticity to bone and a high compressive strength for load-bearing applications in musculoskeletal tissue engineering [141,142,143,144]. Therefore, PCL is more adoptable as a bone substitute biomaterial in tissue engineering and regenerative medicine [145,146,147].

**Figure 2:**
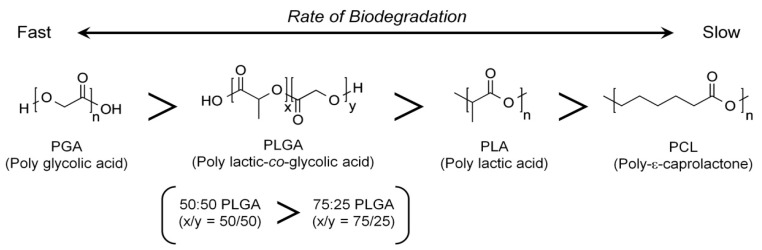
Synthetic biodegradable polymers and the relative hydrolytic degradation rates of biopolymers. PGA hydrolysis is the fastest and PCL is the slowest.

In addition to the mechanical characteristics, PCL has a slower biodegradation rate than other polyester materials due to hydrolytic chain cleavage (hydrolysis) of the aliphatic ester backbone in physiological environments because of the hydrophobic nature by the 5-CH_2_ moieties in the repeat units (Figure 2) [131,139]. The longer biodegradation rate of PCL scaffolds could make spatiotemporal microenvironments during osteogenesis and tissue maturation at defect sites possible within a few years [131].

## 4. Alveolar Bone Regeneration Using the Scaffold Fabrication Technique

### 4.1. Electrospinning for Alveolar Bone Tissue Regeneration

**Figure 3:**
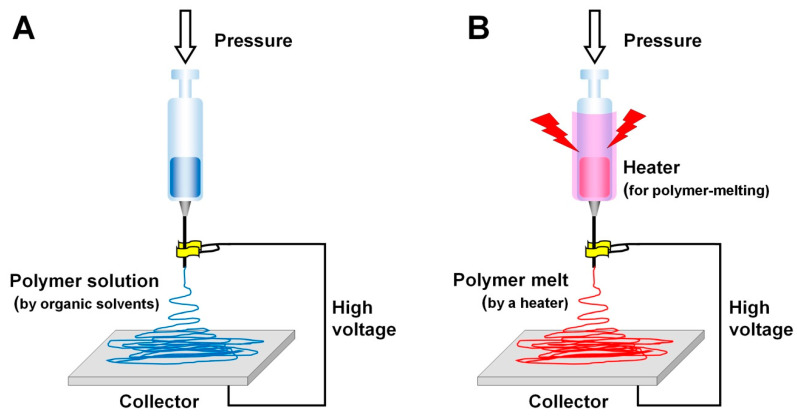
The schematic illustration of electrospinning types: solution electrospinning (**A**) and melt electrospinning (**B**).

The electrospinning technique creates nano-/microfibrous constructs, which mimic ECMs in terms of their structural and morphological properties and so are able to modulate cell-to-material interactions and generate efficient biological responses for tissue morphogenesis [148,149]. Regarding the spatiotemporal arrangements for tissue regeneration using electrospun constructs, the parameters are adjusted to control fiber sizes, porosity, pore interconnectivity, and the surface area of the structures. In Table 1, the typical parameters used in the electrospinning technique are categorized and described, including the environment, the material solution or melt electrospinning, and electrospinning process parameters [149,150,151,152,153]. In spite of such advantages as structure control, ease of fabrication, and the relatively low cost, this technique is limited to manufacturing 3D structures with various micron-scaled fiber dimensions [149,154]. Recently, melt electrospinning fabrication has been developed which avoids using various toxic organic solvents, overcomes the significant dimensional limitations, and allows for the creation of a spatial microstructure with nanofibers and macroscopic pores for bone tissue formation (Figure 3) [155,156,157,158,159].

**Figure 4:**
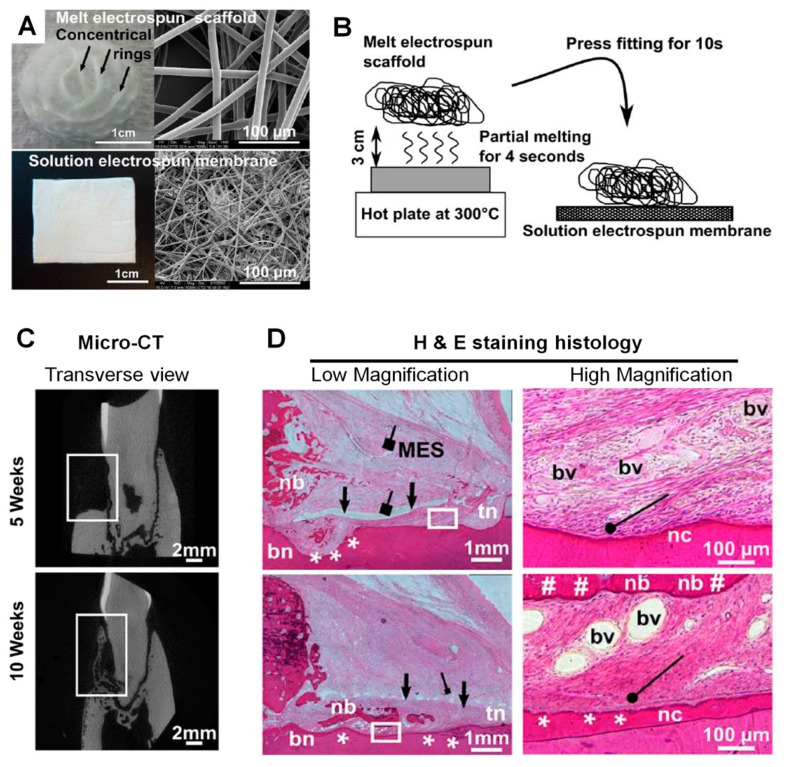
Multipahsic nanofibrous scaffold fabrication. Both polymer solution electrospinning and melt electrospinning membranes were shown with optical images and scanning electron microscopic (SEM) images (**A**). After assembled two different membranes (**B**), the multiphasic scaffolds were transplanted to the periodontal defect sites in the ovine model. (**C**) The micro-computed tomographic (micro-CT) demonstrated the alveolar bone formation with the different time-points (5 weeks and 10 weeks) and (**D**) hematoxylin and eosin (H&E) stained histology showed the mineralized tissue neogenesis around tooth-root surfaces; alveolar bone and cementum. (nb: new bone, tn: top notch, bn: bottom notch, bv: blood vessels, nc: new cementum, MES: melt electrospun scaffold, black arrow: solution electrospun membrane, black square arrowheads: stacked melt electrospun fibers, #: periodontal attachment on newly formed bone, black round arrowhead: oblique PDL fiber insertion, white stars: periodontal regeneration). Adapted with permission from the reference [160].

Vaquette et al. investigated a multiphasic architecture, which was assembled with a solution electrospun membrane, a melt electrospun 3D scaffold, and a multilayered cell sheet construct for hierarchical periodontal complex regeneration [159,160]. The study demonstrated that melt electrospun architectures could provide high porosity with significant interconnectivity [160]. Briefly, two compartmentalized, biphasic PCL scaffolds were manufactured using solution electrospinning with biopolymeric materials in organic solvent for PDL and melt electrospinning for alveolar bone regeneration [160]. After the surgical creation of a periodontal defect to expose tooth-root surfaces in an ovine model, the multilayered scaffolds were transplanted for periodontal complex regeneration [160]. In particular, the melt electrospun fibrous scaffolds had the flexibility to allow high geometric adaptation to periodontal defects and optimize tooth-supporting bone structure formation with specific fibrous connective tissue interfaces [160]. Although cell sheet constructs played a biological role in the periodontal regeneration, the melt electrospun scaffolds allowed osteogenesis and bone ingrowth due to their large interconnective pores, 3D geometries, and nanofibrous topographies [160]. Digital micro-CT and histology images showed that alveolar bone structures were adaptively regenerated on tooth-root surface geometries with a specific thickness for the fibrous connective tissues, i.e., PDLs (Figure 4B,C). In other words, multiphasic nanofibrous scaffolds, which were manufactured and assembled by two different electrospinning methods, significantly contributed to the compartmentalization of multiple tissue formations and controlled, spatiotemporally, the hierarchical architecture dimensions of alveolar bone formation (Figure 4) [160].

### 4.2. 3D Printing Techniques for Alveolar Bone Tissue Regeneration

Regarding bone tissue engineering and regenerative medicine, 3D printing techniques have been actively investigated and 3D structures have been manufactured using various methods, such as fused deposition modeling (FDM) through the extrusion of molten synthetic thermoplastic biomaterials, selective laser sintering (SLS) through the recrystallization of biopolymeric powders using a scanning laser beam, and bioprinting by fabrication with bioink materials including cells, drugs, or biologics [134,161,162,163,164]. After computer-aided design (CAD)-based 3D architectures or medical image-based datasets are created in the stereolithography (STL) file format, which is easily adoptable for different 3D printing systems, selected biopolymeric materials could be printed using layer-by-layer manufacturing systems, and selected with the required parameters, e.g., resolution, feature dimension, biomaterial properties, and regeneration target tissue specificity [13,134,165,166]. Compared with other craniomaxillofacial bone constructs, alveolar bone scaffold design and manufacture as a tooth-supporting tissue remains a challenge due to the structural complexity and geometric adaptability of tooth-root surfaces [2,3,38]. In addition, fibrous connective tissue regions (PDL interfaces with a size of approximately 250–300 µm) should be secured between the alveolar bone and teeth to avoid ankylosis, i.e., the fusion of the bone to the tooth-root surface during alveolar bone regeneration. Therefore, compartmentalized structures for a single scaffold system in the alveolar bone and PDL regions should be designed and manufactured with certain critical requirements, including a manufacturing accuracy and printing resolution appropriate for periodontal hierarchical architectures [1,13,167].

FDM has been simply utilized for microchannel fabrications with dimensional controls, but the low resolution and accuracy limit it with regard to micron-scaled architecture designs for bone tissues as well as angularly organized PDL regeneration [168]. In 2012, a medical image-based periodontal defect-fit scaffolding system was investigated to promote periodontal tissue infiltration and ingrowth to support tooth structures using the 3D wax printing technique [54,166]. Although there were more steps for scaffold fabrication due to casting the PCL biopolymer with sacrificial wax molds, the 3D wax printer provided a high accuracy and resolution for microarchitectures to modulate periodontal tissues. On the basis of this approach, Park recently developed a prototype of a periodontal complex scaffold to be used as the spatial platform for bone–PDL–cementum formation [13]. Using the 3D reconstructed tooth image which was generated using microcomputed tomography (micro-CT) and created in the STL file format, a bone scaffold with a bioactive factor loading space and cementum layer was designed; it also incorporated a PDL-guiding surface with microtopographies and was manufactured using the 3D wax printing system and the PCL solution casting method [6,13,54,166].

**Figure 5:**
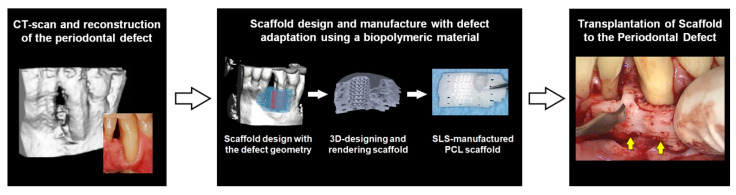
The clinical application for periodontal tissue regeneration using 3D printing system, selective laser sintering (SLS) technique. The scanning the labial defect image in a mandible was utilized to design a periodontal defect-fit scaffold and created by SLS 3D printing system. After sterilizing the scaffold, it was transplanted to the defect sites in the human patient. Adapted with permission from the reference [134].

Rasperini et al. adopted a preclinical concept involving structural compartmentalization for periodontal complex neogenesis using the fenestration defect in a rodent model [166] for clinical applications [134]. On the basis of the cone-beam CT image dataset of a labial periodontal defect, the customized fiber-guiding scaffold was designed and manufactured using the SLS system [134]. Although the preclinical study demonstrated that the 3D wax printing system could create microstructure details in small features, the organic solvents used to remove wax molds (the polymer solution casting method) are not allowed in clinical situations, so the SLS system with the US-FDA-approved PCL biomaterial was exploited for this clinical situation (Figure 5) [134]. Approximately 82% adaptation of the scaffold to the defect geometrywas clinically transplanted, and the site showed no severe clinical findings, such as acute or chronic inflammation, infection, or dehiscence for over 1 year [134,168]. After 14 months, the fragments of the transplanted scaffold were removed and analyzed for mineralized tissue healing and biodegradation by analyzing the molecular weight difference between baseline and at 14-month [134]. Hematoxylin and eosin (H&E) and Masson’s trichrome staining were performed to show tissue attachment to the scaffold matrices and new bone formation within a milieu of primarily granulomatous tissues [134,168].

### 4.3. Biologic Immobilization to Localize Bone Tissue Formation Using the Chemical Vapor Deposition (CVD) Polymerization Technique

As a result of effective tissue regeneration, US-FDA-approved growth factors have been featured as biological factors, such as bone morphogenetic proteins (BMPs) for mineralized tissue formation (BMP-2 and BMP-7) and platelet-derived growth factor (PDGF) for multiple periodontal tissue neogenesis [78,169]. In particular, growth factors can recruit endogenous stem cells, which originate from the tissues around transplanted scaffolds and regulate to directly promote osteogenic differentiation in stem cells [170,171]. Therefore, growth factor (especially, BMPs for bone tissue formation) localization and immobilization with scaffolds have been recently investigated in techniques such as the physical encapsulation/immobilization of growth factors, the absorption of growth factors into scaffolds, and layer-by-layer self-assembly [75,78,169]. Although many studies have reported the efficacy of their growth factor delivery systems in preclinical and clinical situations, the delivery methods are still limited by the potential toxicity of the high doses, the high doses required to overcome biologic loss, and their short half-lives [78,172]. Moreover, they are unpredictable in delivering growth factors for target tissue regeneration at an optimal concentration, and it is still a challenge for various biologics to promote multiple tissue regeneration within complicated microenvironments.

**Figure 6:**
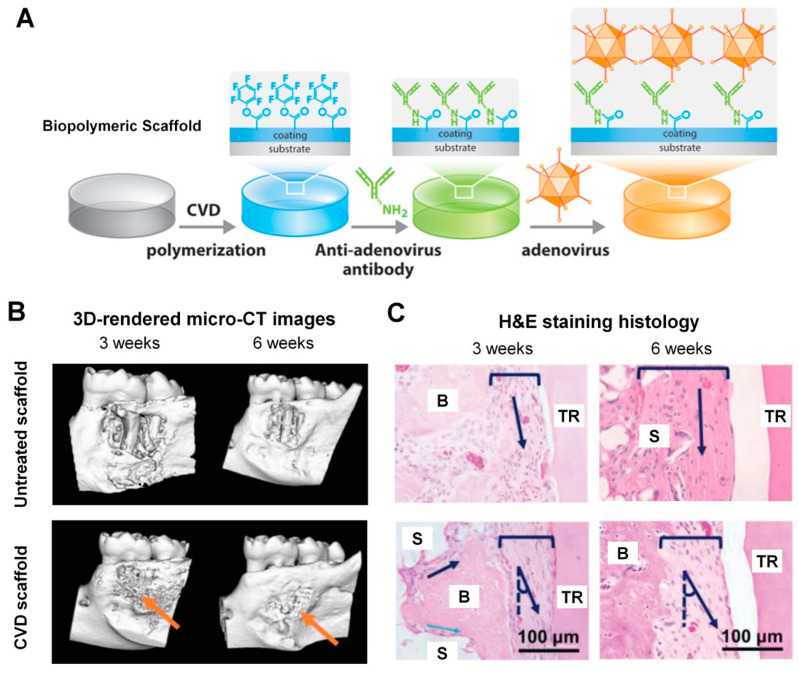
Immobilization of viral vectors for periodontal tissue regeneration. The schematic illustration showed the process of viral vector (adenovirus) attachments on biomaterial surfaces (**A**). Immobilized gene therapy technique promoted alveolar bone formation in the fenestration periodontal defect sites compared with untreated scaffolds. The 3D micro-computed tomography (micro-CT) images could be quantitatively and qualitatively analyzed for the mineralized tissues regeneration with cementum-denuded root coverage (**B**). The hematoxylin & eosin staining histology demonstrated alveolar bone formation around tooth-root surfaces with the secureness of PDL regions (**C**). (B: bone, TR: tooth root, S: scaffold, arrow: orientation of cell nuclei to tooth-root). Adapted with permission from the reference [173,174].

Instead of rapidly degradable growth factors, gene therapies have been recently investigated to immobilize viral vectors on polymeric scaffold surfaces for alveolar bone tissue formation [173,174,175]. In particular, the chemical vapor deposition (CVD) technique can modify FDA-approved biopolymeric material surfaces with chemical conjugations of antibodies, which mediate in the immobilization of viral vectors on the scaffold surfaces [169,173,174,176]. Hao et al. treated three different biomaterial surfaces using the CVD technique to immobilize adenoviral vectors of PDGF-BB (AdPDGF-BB) and BMP-7 (AdBMP-7) for spatially compartmentalized tissue formations [174]. For the covalent immobilization of viral vectors, after a thin polymer layer including pentafluorophenol (PFP)-ester groups was coated on the surfaces of disc-shaped biomaterial substrates using the CVD polymerization technique, an anti-adenovirus antibody was chemically tethered to the coated surfaces (Figure 6) [174,177,178]. The chemically modified substrate surfaces had adenovirus-binding specificity for an antigen–antibody interaction, and covalent immobilization of AdBMP-7, and AdPDGF-BB was successfully verified using X-ray photoelectron spectroscopy (XPS) and immunofluorescence analysis (Figure 6) [174]. To validate the bioactivity of tethered AdBMP-7 and AdPDGF-BB for periodontal tissue engineering, human PDL progenitor cells were seeded onto three different materials. In the results, the transduction efficiency of viral vectors to PDL cells was analyzed by protein expression levels from cell proliferation by AdPDGF-BB and osteogenic differentiation by AdBMP-7 (Figure 6) [174]. Hao et al. demonstrated that fabrication could become the potential candidate method for selecting the bioactivities of different regions in a single scaffold with sustained protein production, more localized target tissue formation, and optimal protein expression in regeneration-required sites [174].

On the basis of the viral vector immobilization technique using the CVD method [174], Pilipchuk et al. developed the customized scaffolding system to induce bone formation at the periodontal fenestration defect, which involved the dimensional complication of controlling the regeneration of the alveolar bone, the tooth-supporting bone structure [173]. After manufacturing random-porous scaffolds, adenoviruses encoding genes for BMP-7 (AdBMP-7) and PDGF (AdPDGF-BB) were chemically tethered onto scaffold surfaces [173]. The microcomputed tomographic images were analyzed for mineralized tissues at the defect sites and adenovirus-immobilized scaffolds significantly promoted newly formed alveolar bone tissues with or without PDL-guiding patterns (Figure 6) [173]. Although there was a limitation regarding optimizing the required concentration of viral vectors for bone regeneration within the rodent fenestration defect of 2 mm × 3 mm for the CVD immobilization technique, the single (AdBMP-7) and dual (AdBMP-7 and AdPDGF-BB) biologic treatment groups interestingly showed the acceleration of bone tissue formation filling defects around tooth structures at 3 weeks (Figure 6) [173]. Using the histological images, the regeneration of tooth-supporting mineralized tissues was assessed and the interfacial tissues (the fibrous connective tissues) were qualitatively analyzed between the bone and tooth surfaces (Figure 6) [173]. In this study, the CVD method provided the selective bioactive surfaces for regionally compartmentalized scaffolds and immobilized the required gene-encoded viral vectors to induce various tissue formations with tissue integration through mineralized tissue formation and deposition.

## 5. Cementum Regeneration Using Scaffold Fabrication

### 5.1. Chemical Fabrications of Fibrin Scaffolds

Cementum of the periodontal complex is the interfacial mineralized tissue between tooth dentin and PDL tissues and plays a pivotal role in facilitating the attachment of PDL fibrous tissue bundles to the tooth-root surface [14,179]. Therefore, cementogenesis (cementum formation) surrounding the tooth surfaces is one of key procedures in periodontal regeneration for the systematic restoration of tooth-supporting structures [180]. Although numerous animal studies using various periodontal regenerative techniques have shown that cementum regeneration is feasible, it is still a challenge to control and predict the spatiotemporal cementum deposition on tooth-root surfaces for the PDL anchorage and Sharpey’s fiber insertion [1,38]. As a result of this complication, some investigations currently use periodontal stem cells to promote cementogenesis by cementogenic differentiations with physiological or pathological responses rather than using developed biomaterial-based engineered platforms [1,181].

**Figure 7:**
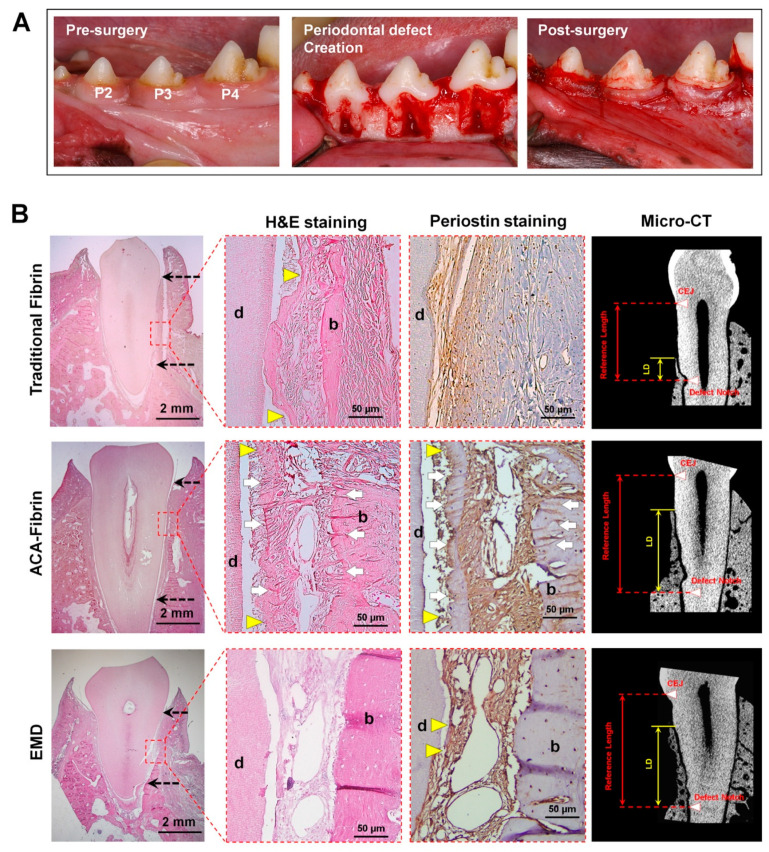
Fibrin fabrication to promote periodontal mineralized tissue formations; osteogenesis and cementogenesis. Using the canine model, the periodontal defects around the tooth-root surfaces were surgically created and biomaterials were transplanted (traditional fibrin, ε-aminocaproic acid (ACA) modified fibrin (ACA-fibrin), and enamel matrix derivative (EMD)) (**A**). For morphological analyses with Sharpey’s fiber insertion, hematoxylin and eosin (H&E) staining and periostin staining methods were utilized (**B**). In particular, cementum regeneration with Sharpey’s fiber anchorages could be significantly found in the ACA-fibrin group even though the alveolar bone formation showed in both ACA-fibrin and EMD groups (**B**). (black dash arrow: surgically created defect, yellow triangle: cementum layer, white arrow: Sharpey’s fiber, d: dentin, b: bone, LD: linear distance of bone regeneration (root coverage), CEJ: cementoenamel junction). Adapted with permission from the reference [14].

Recently, Park et al. recognized that the control of fibrinolysis can regulate and enhance cementogenesis in vitro and in vivo, including functioning restorations of new cementum [14,179]. Because cementoblastic cells in natural fibrin matrices demonstrate higher expression levels of the plasminogen activator, which contributes to fibrinolysis more than osteoblastic cells, cementoblast apoptosis is more rapidly induced by the loss of cementoblast–fibrin (or cell–material) interaction [14]. To improve cementoblast viability in fibrin matrices, the plasminogen inhibitor (ε-aminocaproic acid; ACA) was added to matrices in order to slow down fibrinolysis, which is the process of substrate destruction for cell survival and maintains the polymerized fibrin structures for cementogenic differentiation by cementoblasts [14]. In the results, the chemically modified fibrin (ACA-fibrin) matrices promoted cementogenic differentiation for mineralization and higher expression levels of mineralization-associated molecules in in vitro studies without fibrinolysis [14]. On the basis of this modification, cementum regeneration around the tooth-root surfaces in a canine model was performed with: (1) traditional fibrin; (2) ACA-fibrin; and (3) enamel matrix derivative (EMD) [14]. Immature porcine tooth enamel protein (EMD) is generally known to promote various activities involving periodontal hard tissue formation from preclinical and clinical studies [182,183]. Therefore, EMD has been clinically approved by the US-FDA and is currently commercially available under the name Emdogain^®^ by Institut Straumann AG, Switzerland [184]. In quantification assessments of alveolar bone formations using micro-CT, there was no statistical difference between ACA-fibrin and EMD groups in defect sites (Figure 7); however, ACA-fibrin matrices promoted cementogenesis around the tooth-root surface with histological and morphological differences compared with two other groups (Figure 7) [14]. In particular, anchored Sharpey’s fiber bundles in the PDL interfaces were found around newly formed mineralized tissue surfaces (the alveolar bone and cementum). The connective fibrous tissue insertion between mineralized tissues is typically important to form structural integrations and compartmentalized multiple tissue complexes [14]. Therefore, the chemical control of biomaterial degradation could regulate the formation of the micron-scaled mineralized layer (cementogenesis) and spatially facilitate specific multiple periodontal tissue formations without ankylosis [14].

### 5.2. Biomimetic Cementum Fabrication Using Collagen Lamella Constructs

**Figure 8:**
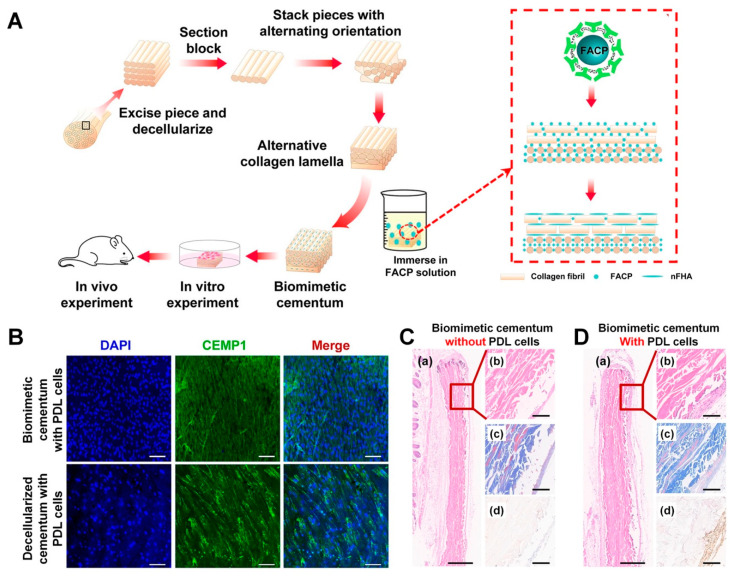
The biomimetic cementum construction with fluorine, which could catalyzed biomineral formation in in-vitro and in-vivo. The schematic illustration demonstrated Bioskiving process with fluorine containment for biomineralization and the constructs of biomimetic cementum were examined in in-vitro and in-vivo (**A**). In in-vitro experiments, two different constructs (biomimetic cementum and decellularized human cementum) had similar expression levels of CEMP-1 after seeding periodontal ligament (PDL) cells (**B**). In-vivo study showed that biomimetic cementum structure could stimulate cementogenic differentiation of PDL cells and promote cementogenesis to form mineralized layer formation (**C-d**,**D-d**). Hematoxylin and eosin (H&E) staining analyzed tissue morphologies with two different magnifications (low magnification (**C-a**,**D-a**) and high magnifications (**C-b**,**D-b**)). Masson trichrome staining was used for collagen and bone ((**C-c**,**D-c**)). Immunohistochemical analysis was qualitatively analyzed for cementogenesis using CEMP-1 (**C-d**,**D-d**). Scale bar: 100 μm. Adapted with permission from the reference [185].

In contrast to other mineralized tissues, cementum has a high concentration of fluoride, which is a great potential candidate as a catalysis to contribute to tissue mineralization and to accelerate cementum regeneration through mineral deposition on tooth-root surfaces [185]. Recently, Yang et al. developed collagen scaffolds with fluorine-contained amorphous calcium phosphates (FACP) to promote mineralized tissue formation on tooth-root surfaces with specific micron-scaled dimensions [185]. To create similar hierarchical constructs to cementum with unique alternating collagen lamella (ACL) structures, the FACP–collagen scaffold was fabricated using the Bioskiving process, which is a newly developed method for the 3D scaffolding system using decellularized tendon tissues [185]. In particular, the technique can three-dimensionally organize the nano-/microstructures of collagen constructs through a combination of processes, such as sectioning tendon-derived (tenton-decellularized) structures and stacking sheets with alternating orientations of collagen lamellae (Figure 8) [185,186].

The Bioskiving process fabricates ACL microarchitectures to mimic the hierarchical twisted structures of the collagen matrix in cementum [25,187] by controlling the thickness of the constructs with specifically rotated collagen lamellae [185]. In one study, after ACL scaffolds were immersed in FACP solution to form biomimetic cementum constructs, biological examinations were designed and performed to validate various cell activities, such as attachment, proliferation, differentiation in in-vitro and cementum-like formation, with a high expression level of CEMP-1, the cementogenic differentiation marker in in vivo (Figure 8) [185]. Because this study showed that biomimetic cementum structures with human PDL cells were found subcutaneously in immunodeficient mice, it was not demonstrated that the biomineralized collagen scaffold fabricated using the Bioskiving method was able to create a cementum layer on the tooth-root surface and tissue integration with the tooth dentin surface. However, the technique was able to mimic sophisticated hierarchies of human cementum with rotated-stacked angular microstructures and promoted mineralized tissue formation in physiological microenvironments [185].

## 6. Prospective Strategies for Periodontal Hard Tissue Formations

To date, many studies in periodontal tissue engineering have focused on compartmentalizing and regenerating individual periodontal tissues (alveolar bone, PDL, or cementum) for the specific dimensions of spatial interfaces using biopolymeric materials. In particular, the biopolymer fabrication techniques described in this review can promote mineralized tissue formation with the spatiotemporal regulation of tissue ingrowth into periodontal defects using their geometries, biologic immobilization, degradation controls by chemical modification, and manufacturing methods for 3D architectures. Nevertheless, it remains a challenge to achieve multiple tissue integration between mineralized tissue and fibrous connective tissue bundles, which is the ultimate goal of interfacial periodontal tissue formation. For the restoration of the regenerated periodontal complex, fibrous tissue calcification and its neogenic regulation within the specific zone is important as to enhance biomechanical integration for the hard-to-soft tissue complex, with engineered Sharpey’s fibers acting as the tooth-supporting structure. In addition to multiple tissue integration, understanding the relationship between reconstructed hierarchical structures and integrated tissue, functioning with the biomechanical properties of the alveolar bone–PDL–cementum complexes, is of vital importance. Therefore, the future strategies of interest are multidisciplinary in nature and involve approaches such as the regulation of calcified interfacial tissue morphogenesis, the biochemical interactions of cell–tissue materials, scaffold architecture designs for spatiotemporal arrangements of multiple tissue growth, and the functioning restoration of the engineered periodontium tooth with integration.

## Figures and Tables

**Table 1:** Various electrospinning parameters were described. The parameters should be significantly considered because they could affect electrospun fibrous product qualities.

Environment Parameters	Material (Polymer Solution or Melt) Parameters	Electrospinning Process Parameters
• Humidity • Temperature	• Concentration of polymer solution • Properties of polymer solution or melt • Molecular characters ✓ Molecular weight ✓ Polymer conductivity	• Distance of needle-collector • Needle diameters • Electric field by voltage • Flow rate • Collectors ✓ Plate type ✓ Drum (rotating) type

## Abbreviations

PDL	Periodontal ligament
CEJ	Cementoenamel junction
3D	3-dimensional
CAD	Computer-aided design
ECM	Extracellular matrix
3D	Three-dimensional
US-FDA	US Food and Drug Administration
PGA	Poly glycolic acid
PLA	Poly lactic acid
PLGA	Poly lactic-co-glycolic acid
GBR	Guided bone regeneration
PCL	Poly-ε-caprolactone
FDM	Fused deposition modeling
SLS	Selective laser sintering
STL	Stereolithography
H&E	Hematoxylin and eosin
BMP	Bone morphogenetic proteins
PDGF	Platelet-derived growth factor
CVD	Chemical vapor deposition
AdPDGF-BB	Adenoviral vectors of PDGF-BB
AdBMP-7	Adenoviral vectors of BMP-7
PFP	Pentafluorophenol
XPS	X-ray photoelectron spectroscopy
ACA	ε-aminocaproic acid
EMD	Enamel matrix derivative
FACP	Fluorine-contained amorphous calcium phosphates
ACL	Alternating collagen lamella
CEMP-1	Cementoblastoma-derived protein 1
